# Supplementary material for: Four Futures for Occupational Safety and Health
Source: Int J Environ Res Public Health. 2023 Feb 28;20(5):4333. doi: 10.3390/ijerph20054333 (PMC10001522; doi:10.3390/ijerph20054333)
Supplement: Supplementary file 1 [file ijerph-20-04333-s001.zip › ijerph-2228366-supplementary.pdf]

Supplementary Materials

Four Futures for Occupational Safety and Health

Sarah A. Felknor, Jessica M. K. Streit, Nicole T. Edwards, and John Howard

File S1: Trends, Issues, Plans, and Projections (TIPPS), with supporting references. Full citations are available in the References section of the main manuscript.

OSH Activities

- **Changing nature of work:** Changes in work organization (non-standard and precarious employment) are increasing [57].
- **Increasing telework:** Remote/telework has gained acceptance in multiple industries and is increasing [58].
- **Incorporation of tech:** AI and machine learning are increasingly being used to identify worker health and safety issues in large disparate datasets – some containing personal information [59].
- **A rise in holistic worker health:** A more holistic model of worker health is emerging, including integrating worker well-being into the workplace will influence the research and practice of OSH [6,57,60,61].
- **New industry exposures:** New industries from manufacturing methods (3D, metals additive manufacturing) and the green economy (battery development, renewable energy, and recycling) are impacting worker population and exposures [57,58].

Facilities

- **Flexible workspaces:** Flexible and co-working spaces are the fastest-growing type of office space and are expected to make up 30% of commercial real estate by 2030 [62].
- **Increasing lab automation:** Automation and robotics in laboratories are incrementally increasing over time. The global lab automation market is expected to experience an annual growth rate of 5% from 2018 ($3.14 billion) to 2025 ($4.64 billion) [63].
- **Lab work requires less lab time:** Tech advances (automation, AI, and data processing) are reducing the number of hours scientists spend in labs, thereby increasing the time they can spend focused on research design, analyses, and reporting [64].
- **Office work is making us sick:** The office workstation is fast becoming one of the most dangerous places to work. Sedentary, screen-based office work is associated with an increasing number of adverse physical and mental health outcomes, to the point where some researchers believe the Fourth Industrial Revolution may leave us all permanently sick [65].

Policies

- **Global leader influences:** Three trends are threatening the U.S. position as a global leader in science and engineering: talent shortage, physical and IT infrastructure, and uncoordinated partnerships [66].
- **New hiring practices:** New hiring practices (e.g., sub-contracted or temporary) are resulting in jobs that have fewer or no benefits [67].
- **Biometrics and privacy:** Biometrics are being used for government surveillance, raising concerns about privacy [68].
- **Increasing mental and behavioral health issues:** Frequency and severity of formally documented work-related mental and behavioral health issues is increasing [69,70].
- **Investment in IT modernization:** Federal agencies are dedicating more resources to the development and sustainment of cloud technologies and IT modernization [71].

- **Tracking and monitoring for COVID-19:** COVID-19 continues to drive new employee tracking/monitoring practices (e.g., employee productivity tracking). As the reopening process continues, the scale and types of data collected by employers to combat the threat of COVID-19 will continue to increase [72,73].
- **Developing social credit systems:** A social credit scoring system similar to the one employed by the Chinese government is organically developing in the U.S., driven by technology-industry user policies and surveillance of social media activity by private companies [74].
- **Supporting mental health and well-being:** Employers continue to address mental health/well-being challenges by offering programs, applications, workshops, etc. that help workers monitor and manage stress levels, sleep, relaxation, and emotional resilience [75].

Resources

- **Wages**: Global wages are growing more slowly [76].
- **Workforce Composition:** Currently, 40% of the U.S. working population are considered contractors, and that number is increasing [76]. Over a quarter of the world's largest employers are renting workers as their primary business [77,78].
- **Workforce Demographics**: Overall U.S. population and workforce growth are declining. Labor participation rates for young and older workers are notably decreasing, but certain segments (e.g., the Hispanic labor force) are growing [79].
- **Digital Communication**: The demand for digital communication platforms for business and personal use is increasing, and with it the need for secure end-to-end encryption of billions of messages per day [80].

Workforce

- **Aging population:** The U.S. population (and thus its workforce) is aging [81].
- **Increased working life:** As people are living longer, then are expected to work longer. The pressure comes from many sources, including governments and individual workers themselves [82].
- **Uptick in anti-aging research:** Bio-tech companies are raising investment capital for anti-aging research, and careers may become much longer as a result [83].
- **Federal staffing shortfalls:** The Federal workforce is failing to keep up with the private sector as it relates to capabilities of staff [84].
- **Steady OSH employment growth:** Overall employment of OSH specialists and technicians is expected to grow 4 percent from 2019 to 2029, about as fast as the average for all occupations [85].
- **Shrinking OSH workforce opportunities:** Aspiring OSH professionals are struggling to find work amid shrinking safety and health career opportunities resulting from industrial improvements and outsourcing [86,87].
- **Shifting education paradigms:** Alternatives to traditional college degrees are gaining acceptance across industries and employers, including the Department of Labor [88].
- **Evolving HR priorities:** Nine HR trends will continue to emerge as the result of pandemic disruption to work, generating HR challenges and shifting HR priorities [89].
- **The rise of small remote teams:** Automated system implementation is causing businesses to operate in smaller teams while also transitioning into remote work [90].

Issues

OSH Activities

- **Organize v. Individualize:** Will gig workers organize, or will they continue to fend for themselves at the individual level? [67]
- **Leading v. Lagging regulations:** Will worker safety and health regulations keep up with rapid changes in workplace, or will OSH regulation lag behind changes in industry? [91,92]

- **Traditional v. New sectors:** Will traditional industry sectors continue into the future, or will there be new industrial sectors that introduce new workplace exposures and risks? [92]
- **Robots assist v. Robots endanger:** Will robotics assist workers in the future, or will their use increase risk of work-related injuries? [91]
- **Increased v. Decreased job opportunities due to automation:** Will job opportunities continue to increase in the future, or will automation significantly reduce the number and type of employment available? [92]

Facilities

- **Physical facilities v. Virtualization:** Will technology be incorporated into existing worksites, or will it make physical facilities obsolete? [93-96]
- **Face-to-face v. Virtual collaboration:** Will physical facilities integrate virtual work policies and practices, or will there be a dichotomy of 'face-to-face' and 'virtual' workplaces? [97,98]
- **Technology as lab equipment v. Technology as lab coworker:** Will technology's role in science labs of the future stop at equipment and instrumentation, or will cobots work alongside laboratorians? [99,100]

Policies

- **Risk-based v. Rights-based policies:** Will risk-based or rights-based (e.g., privacy) policies dominate as surveillance and wearable technologies advance [101]?
- **Surveillance increases productivity v. Surveillance erodes trust:** Will workplace policies regarding the prevalence and normalization of surveillance technology to track employee activity have more implications for productivity or the erosion of employee trust [102]?
- **Social credit takes hold v. Social credit derailed by privacy:** Will social credit scores impact companies, employment, and health and safety-related incentives and behaviors, or will privacy demands prevent the system from taking root in the U.S. [103]?
- **Stagnant v. Increased public health funding:** Will historically inadequate levels of public health resourcing continue to persist following the COVID-19 experience, or will there be an influx of funding post-pandemic [104]?
- **HR policies continue v. HR policy evolution:** Will OPM continue its current hiring and retention practices, or will policies and incentives evolve as hiring practices shift in the nation [105]?
- **Continuation v. Evolution of research priorities:** Will NIOSH research priorities remain consistent with the 2019-2024 Strategic Plan, or will the growing policies and costs associated with mental health-related issues (e.g., drug use, PTSD) drive the need for NIOSH to further expand its expertise in psychosocial factors [69]?
- **Research v. IT as leading driver:** Will research and service activities influence the evolution of IT and data governance in OSH, or will the increased use of cloud technology in the federal government dictate for the way we approach research and service functions [71]?

Resources

- **Etiologic v. Intervention research as priority:** Will OSH research focus on identifying new risks and hazards in the future of work, or will resources shift to evaluating the implementation of OSH solutions? [57]
- **Proactive v. Reactive OSH:** Will OSH be incorporated in a way that ensures new technology implementation avoids unintended consequences, of will OSH remain reactive to the changes that occur? [106]
- **Privacy maintained v. Privacy compromised:** Will we balance the increase in access and data that technology brings while maintaining privacy and security, or will privacy and security be compromised? [106,107]
- **Data access facilitated v. Data access limited:** Will we be able to balance the needs for data security with data access for research purposes, or will data access for research remain limited in the face of privacy concerns? [108-111]
- **Traditional v. New methods for worker access:** Will OSH researchers remain reliant on employers when it comes to collecting data from workers, or will OSH shift to new worker access approaches? [57]

- **Peer review as the standard v. Legitimizing alternative sources of information:** Will the growing range of diversified research outputs, which can drive research towards better practice, be universally accepted in the future, or will peer-review publications remain the standard? [112]
- **Research collaboration v. Research isolation:** Will university-industry partnerships solve the big challenges facing society, or will academia retain its 'ivory tower' reputation? [113]
- Will federal agencies (including OSHA, BLS, and NIOSH) and state health departments continue to work in parallel, or will they develop a collaborative system of OSH surveillance data collection, analysis and reporting? [114]
- **Technology improves data collection v. Technology complicates research:** Will advances in tools and technologies—sensor systems, analytic methods, molecular technologies, computational tools, and bioinformatics—complicate or improve the collection of exposure science information? [115]
- **Problem-defining v. Problem solving:** Will OSH continue to operate within a problem-defining paradigm, or will data interoperability and open data movements allow the field to move to a problem-solving paradigm? [116]
- **Individual identity v. Collective benefits:** How can individuals maintain their sense of our own self as being sacred in some ways--with their individual data protected--while at the same time enjoying the benefits of policies, predictions, and decisions only possible through the collective power of pooling individual data throughout society? [117]
- **Mitigation v. Exacerbation of risk by AI:** Will AI mitigate or exacerbate risks in existing digital work environments? [118]
- **Complex v. Streamlined public health:** As the nature of work and employment arrangements continue to change and diversify, will the U.S. public health system become more complicated to navigate, or is a fully digital and portable public health system possible? [119]

Workforce

- **Talent retention v. Talent loss:** Will federal agencies be able to attract and retain young talent, or will private industry win the talent acquisition war? [120]
- **Education continues v. Education reform:** Will pre-COVID professional preparation approaches be sufficient in the future, or will the system radically change? [121]

Plans

- **The U.S. National Institute of Standards and Technology (NIST)** aims to make virtual reality simulations a new safety training and testing tool, focusing initially on first responders [122].
- **The U.S. Environmental Protection Agency (EPA)** plans to phase out all mammal testing by 2035, which opens the door for new nonanimal labs and models [123].
- **The U.S. Department of Defense (DOD)** aims to expand information sharing capabilities by 2030 to allow high-performance electronic connectivity that flexibly and reliably links computers and systems, manned and unmanned, of different types and generations, which will enable greater use of AI and IoTs in federal workplaces [124].
- **The United Nations Development Group (UNDG)** sets out general guidance on data privacy, data protection and data ethics for concerning the use of big data, collected in real time by private sector entities as part of their business offerings, and shared with UNDG members [125].
- **The National Safety Council (NSC)** launches program to eliminate death on the job by the year 2050 [126].

Projections

OSH Activities

- **Workforce of the Future: The Competing Forces Shaping 2030:** PricewaterhouseCoopers anticipates the future of work will be shaped by five megatrends: technological breakthroughs, demographic shifts, rapid urbanization, shifts in global economic power, and resources scarcity and climate change [127].
- **Technology is Changing the Future of Work. Are you Ready?** Deloitte reports suggest the future of work will be shaped by two powerful forces: the growing adoption of artificial intelligence in the workplace, and the expansion of the workforce to include both on- and off-balance-sheet talent [128-130].

Facilities

- **Top 5 Designers Imagine the Workplace of 2040**: Work culture, the nature of people management, artificial intelligence and data, and even global warming will affect the office environment of the future. The relationship between working, living, and playing will become more fluid. AI will help translate complex relationships–the cultural and operational DNA of organizations–into dynamic, data-driven connections with the built environment [131].
- **What Will the Future of the Laboratory Look Like?** Lab space will be light, lean, and modular. The interaction between the lab worker and the lab will be enabled by virtual systems and augmented reality. Integration of Laboratory Management Systems will cover supply chain, functions, documentation, and processes and can also be connected to big data analytical systems [132,133].
- **4 Life-Changing Technologies We'll See by the Year 2040:** Brain-machine interfaces will make things like social media and surfing the internet hands-free. Machine intelligence could surpass humans in intelligence by 2030 and brain-machine interfaces will help humans keep up. Quantum computers will be more common and may even replace the transistor-based computers. AI will remotely manage hundreds of buildings and security systems, including robotics, intrusions systems, and countermeasures [134].
- **Renovating Research Laboratories for Zero Carbon by 2030:** Architecture 2030 issues challenge for all new and major buildings, especially research buildings (which use more energy), to be carbon neutral by 2030 [135].

Policies

- **Federal Spurt in Cloud Spending Will Extend Well into the Future:** Deltek's analyses predict annual federal spending on cloud computing will grow from the $6.8 billion level in 2020 to $7.3 billion in 2021 and to $7.8 billion in 2022. The investment has underscored the need for increased telework during COVID-19 and will likely support the continued trend of telecommuting by a majority of the workforce [71].
- **Global Risks 2035 Update: Decline or New Renaissance?** The Atlantic Council speculates climate change policies will drive the increased use of alternative power (e.g., nuclear), which will require a needs and hazard assessment for workers constructing these facilities, demolishing old facilities, and operating these new facilities in the age of AI [136].
- **Vision 2030:** The National Science Board anticipates China will invest over $200 billion a year more in R&D than the U.S. by 2030 [66].
- **U.S. Government Science and Technology Strategies:** Reports from the U.S. Air Force predict rapid growth in technology capabilities in response to intensified global competition (e.g., space race and military investments, which will ultimately revolutionize the workplace once these technologies are adapted for commercial applications and adopted in the workplace [124,137].
- **The Future of Safety:** According to a panel of experts, safety professionals will be more involved in workforce planning and policies related to employee training and alternate work situations and there may be a larger focus on the "health" part of safety and health post-pandemic [138].

Resources

- **Shifting Demographics and Social Change:** 3M expects workers in more developed, aging societies will face delayed pension income, stressed pension systems, and gaps between what workers currently save and what they need for retirement [76].
- **What Will the Future of the Internet Look Like?** A Pew Research Center canvass of 530 experts indicates technology will change how we interact and communicate through an increase in virtual reality and other new forms of communication [139].
- **The Future of Big Data:** Experts predict the future of big data will change everything across industries by 2025, and the overwhelming size of big data will create challenges, including privacy and security risks [136].
- **Forecasting Emerging Technologies' Impact on Work:** Forecasts from Dell Technologies suggest emerging technologies will impact the future of human-machine partnerships, and shifts in inclusive talent, empowered workers, and AI fluency will dominate the next decade [141].
- **3 Scenarios for the Future of Research:** Experts at the American Association for the Advancement of Science (AAAS) imagine changes in how research will be created and exchanged by 2029 will be driven by open science, technology, and the rise of China's research investments [142].
- **What Will the Future of Work be Like by 2050?** Russian multinational cybersecurity firm Kapersky projects robots and AI will power the future of work by 2025. Widespread development and use of robots and AI at work will drive legal framework to protect and regulate them while personal assistants perform administrative tasks and psychological issues and communication difficulties increase, scanners and sensors transform digital security, and human work arrangements become even more flexible and fun as repetitive tasks are assigned to robots [143].
- **Employment Projections in a Pandemic Environment**: Scenarios produced by the U.S. BLS provide projections about changes to occupational staffing patterns due to changes in consumer and firm behavior [144].

Workforce

- **4 Bold Predictions for the Workforce of 2030:** Futurist Shannon Gaydos predicts four trends we can expect to see by 2030: a significant drop in traditional four-year degree program enrollment, income share agreements between employers and employees, the rise of digital personal applications to harvest memories, and the use of resignation bonuses to force early organizational departures [145].

Table S1. Glossary of acronyms

Abbreviation	Description
9/11	September 11, 2001 al-Qaeda terrorist attacks on the U.S.
AAAS	American Association for the Advancement of Science
AAOHN	American Association of Occupational Health Nurses
ACOEM	American College of Occupational and Environmental Medicine
AFGE	American Federation of Government Employees
AFL-CIO	American Federation of Labor and Congress of Industrial Organizations
AFT	American Federation of Teachers
AI	Artificial Intelligence
AIHA	American Industrial Hygiene Association
AOHP	Association of Occupational Health Professionals in Healthcare
APA	American Psychological Association
ASSP	American Society of Safety Professionals
BLS	U.S. Bureau of Labor Statistics
BNI	Burden, Need, and Impact
CDC	U.S. Centers for Disease Control and Prevention
COVID-19	Coronavirus Disease of 2019
CSTE	Council of State and Territorial Epidemiologists
DC	District of Columbia
DOD	U.S. Department of Defense
DOL	U.S. Department of Labor
DOT	U.S. Department of Transportation
EPA	U.S. Environmental Protection Agency
H1	Horizon 1
H2	Horizon 2
H3	Horizon 3
HQ	Headquarters
HFES	Human Factors and Ergonomics Society
HR	Human Resources
IFTF	Institute for the Future
IARC	International Agency for Research on Cancer
ILO	International Labour Organization
IoTs	The Internet of Things
IPA	Intergovernmental Personnel Act
ISRP	International Society for Respiratory Protection
IT	Information Technology
MSHA	Mine Safety and Health Administration
NCI	U.S. National Cancer Institute
NCIPC	U.S. National Center for Injury Prevention and Control
NGO	Non-governmental organization
NIEHS	U.S. National Institute of Environmental Health Sciences
NIH	U.S. National Institutes of Health
NIOSH	U.S. National Institute for Occupational Safety and Health
NIST	U.S. National Institute of Standards and Technology
NORA	National Occupational Research Agenda
NSC	National Safety Council
NSF	U.S. National Science Foundation
OH	Ohio
OMB	U.S. Office of Management and Budget
OSH	Occupational Safety and Health
OSHA	Occupational Safety and Health Administration

OSHQ	Occupational Safety and Health Quality
PII	Personally Identifiable Information
PRA	Paperwork Reduction Act
PTSD	Post-traumatic stress disorder
R&D	Research and Development
SEIU	Service Employees International Union
SOHP	Society for Occupational Health Psychology
SRA	Society for Risk Analysis
STEEP	Social, Technological, Environmental, Economic, and Political
TIPPS	Trends, Issues, Plans, and Projections
U.S.	United States
UNDG	United Nations Development Group
USCG	U.S. Coast Guard
WHO	World Health Organization
WTO	World Trade Organization

Table S2. Map of eight drivers of change into each archetypal future

	Continuation	Collapse	New Equilibrium	Transformation
	**Present trends and forces continue without any major disruptions or surprises. The system continues along its current trajectory.**	**The system 'breaks' or falls into a state of dysfunction. The established way of doing things no longer works.**	**The system is confronted with a major challenge. It is forced to adapt in order to 'save itself' and keep the basic structure intact.**	**There is a fundamental change to the system. The rules of the game are scrapped and new ways of doing things emerge.**
**Advanced Technology**	Advances in technology and AI dramatically increase productivity. Reliance on these new technologies outpace the rates at which workers can be retrained and OSH systems can be built to address hazards created by these new technologies.	Automation and robotics replace all manual jobs, leaving fewer opportunities for work. Distrust in AI and electronic surveillance leads to communities of people living with limited use of technology.	The implementation of advanced tech in the workplace is constrained by energy use regulations and notable workforce skills gaps. Organizations focus on human-machine collaboration to maximize outputs.	Improving AI and robotics combine to create unparalleled capacity in analysis and assembly. Human labor is only required for social and artisanal tasks or coordinating AIs in novel ways. Cybernetics, advanced biotechnology, and genetic engineering can augment workers to be more capable, long-lived, and less prone to malady.
**Climate and Energy**	Companies incorporate green policies and increase the use of alternative energies. The rate of adoption of sustainable practices continues to fall short of achieving significant mitigation of climate change.	Humans failed to make the changes needed to slow climate change. Worker exposures to climate changes elude effective OSH prevention measures.	The focus shifts from developing alternative energies to limiting energy use, which impacts work in the energy sector and presents new challenges for OSH innovation and efficacy.	Total worker/total environment health is expected.
**Data Security**	Standardized interoperability methods increase the speed data can travel and exposes complex system vulnerability. Demand drives growth in cybersecurity and worker data protections.	Failure to install adequate online database protection results in hacking of PII and biometric data and a loss of expert credibility. Lack of trust, fear of being monitored, and low data transparency limit opportunities for OSH research and practice.	Increased investments support the development of effective built-in data security systems. While users must adapt to changing conditions, they largely gain through improved data security, linkages, and efficiency.	Data and data systems become integrated with individual workers' identities and their ability to function. Protecting worker data is as important as ensuring worker safety and health.
**Knowledge Generation**	Lack of trust in government agencies and widespread mis and disinformation challenges the ability of research agencies to disseminate accurate health and safety information.	Mis- and dis-information abound as trust in government further deteriorates and authority decentralizes. This creates insurmountable challenges for communicating scientific evidence to improve working conditions.	OSH resources are strained by competing needs. New remote data collection techniques must be developed and evaluated as additional investments are required to enhance OSH information dissemination capacity.	Knowledge generation is driven by population need and research that is effectively implemented with broad benefit is valued.

**Social Credit**	Concern for the (mis)use of social credit scores stalls interest in accepting a monitoring system with access to unregulated personal data by third parties that is used to punish or reward individuals and companies.	Reliance on social credit scores as the "truth standard" for employees and organizations significantly diminishes the role of research in protecting and promoting to worker safety and health.	Employers feel increased pressure to demonstrate and document social responsibility in order to attract and retain top talent.	Social credit scores are a new incentive structure for OSH. Independent credentialling entities create searchable and verifiable OSH quality (OSHQ) scores and companies with high OSHQ scores are sought after by investors and workers.
**Virtual Workplace**	The boundaries between work and personal life are further blurred with advanced communication and data channels. Workplaces, defined by function, are ubiquitous and OSH is challenged to protect workers in virtual environments.	Demand for improved technology far outpaces the supply, making virtual work largely unaffordable. The OSH system weakens as it bifurcates to address concurrent demands for safe and healthy work in both virtual and physical work settings.	Employers pare down central facilities as they adopt remote work practices. OSH becomes responsible for developing valid assessments of 'remote workability' for jobs and workers.	Almost all work is done remotely, and industry sectors are forced to evolve as goods and services are created and delivered virtually.
**Work Arrangements**	Nonstandard work arrangements become more mainstream, and employers embrace hybrid environments that favor the individual over the collective.	The growing complexity of unstructured and fluid work arrangements eventually becomes unsustainable. Workplaces shut down, forcing "survival of the fittest" for organizations and workers.	Workers manage their own careers, benefit portfolios, and risks as they frequently move between nonstandard employers. OSH struggles to study and mange hazardous exposures.	Working 9-5 is obsolete. Employers expand flexible arrangements to compete for workers. Existing performance standards are scrapped as work becomes valued by its contribution to the bottom line.
**Workforce**	Economic trends, individual preferences, and competing priorities result in delayed retirement and an extended working-life continuum. Workers seek to upskill or reskill to stay competitive in the workforce.	Workers face chronic underemployment or unemployment due to job scarcity, increasing mental and physical health issues. Public service systems are unable to meet demand, resulting in limited access to private sector delivery of services.	The implementation of health-supporting initiatives like Total Worker Health® is challenged by a multicultural, international, mobile, and remote workforce. Diversity and inclusion are prioritized for reskilling and upskilling opportunities.	The workforce is more distributed and flexible with multiple and global employers. OSH has expanded to address worker mental health challenges associated with complexity of interfacing with advanced technologies. Workers unable to adjust to these changes depend on Universal Basic Income.
